# Community-Acquired Pneumonia Due to Pandemic A(H1N1)2009 Influenzavirus and Methicillin Resistant *Staphylococcus aureus* Co-Infection

**DOI:** 10.1371/journal.pone.0008705

**Published:** 2010-01-14

**Authors:** Ronan J. Murray, James O. Robinson, Jodi N. White, Frank Hughes, Geoffrey W. Coombs, Julie C. Pearson, Hui-Leen Tan, Glenys Chidlow, Simon Williams, Keryn J. Christiansen, David W. Smith

**Affiliations:** 1 Division of Microbiology and Infectious Diseases, PathWest Laboratory Medicine WA, Queen Elizabeth II Medical Centre, Perth, Western Australia, Australia; 2 Department of Microbiology and Infectious Diseases, PathWest Laboratory Medicine WA, Royal Perth Hospital, Perth, Western Australia, Australia; 3 Division of Forensic Pathology, PathWest Laboratory Medicine WA, Queen Elizabeth II Medical Centre, Perth, Western Australia, Australia; National Institutes of Health, United States of America

## Abstract

**Background:**

Bacterial pneumonia is a well described complication of influenza. In recent years, community-onset methicillin-resistant *Staphylococcus aureus* (cMRSA) infection has emerged as a contributor to morbidity and mortality in patients with influenza. Since the emergence and rapid dissemination of pandemic A(H1N1)2009 influenzavirus in April 2009, initial descriptions of the clinical features of patients hospitalized with pneumonia have contained few details of patients with bacterial co-infection.

**Methodology/Principal Findings:**

Patients with community–acquired pneumonia (CAP) caused by co-infection with pandemic A(H1N1)2009 influenzavirus and cMRSA were prospectively identified at two tertiary hospitals in one Australian city during July to September 2009, the period of intense influenza activity in our region. Detailed characterization of the cMRSA isolates was performed. 252 patients with pandemic A(H1N1)2009 influenzavirus infection were admitted at the two sites during the period of study. Three cases of CAP due to pandemic A(H1N1)2009/cMRSA co-infection were identified. The clinical features of these patients were typical of those with *S. aureus* co-infection or sequential infection following influenza. The 3 patients received appropriate empiric therapy for influenza, but inappropriate empiric therapy for cMRSA infection; all 3 survived. In addition, 2 fatal cases of CAP caused by pandemic A(H1N1)2009/cMRSA co-infection were identified on post–mortem examination. The cMRSA infections were caused by three different cMRSA clones, only one of which contained genes for Panton-Valentine Leukocidin (PVL).

**Conclusions/Significance:**

Clinicians managing patients with pandemic A(H1N1)2009 influenzavirus infection should be alert to the possibility of co-infection or sequential infection with virulent, antimicrobial-resistant bacterial pathogens such as cMRSA. PVL toxin is not necessary for the development of cMRSA pneumonia in the setting of pandemic A( H1N1) 2009 influenzavirus co-infection.

## Introduction

Influenza is an important cause of morbidity and mortality [1]. Bacterial co-infection is an important contributor to morbidity and mortality during influenza pandemics [2], [3] and during periods of seasonal influenza activity in inter-pandemic periods [4]. In particular, *Staphylococcus aureus* pneumonia is a well-described life-threatening complication of pandemic [5], [6] and seasonal [7]–[10] influenza. Following the recent worldwide emergence of community-onset methicillin-resistant *S. aureus* (cMRSA), several reports have described severe co-infection with seasonal influenzavirus and cMRSA [7]–[12]. Severe necrotizing *S. aureus* pneumonia in previously healthy individuals (often with a preceding history of influenza-like illness[ILI]) has been associated with the synergohymenotrophic exotoxin Panton-Valentine Leukocidin (PVL) [11]–[13], and the genes encoding PVL are present in many cMRSA clones, including ST8-MRSA-IV/USA300 in the United States [14], ST59-MRSA-V_T_ in Eastern Asia [15], ST80-MRSA-IV in Europe [16], and ST93-MRSA-IV and ST30-MRSA-IV in Australia [17].

In April 2009 pandemic A(H1N1)2009 influenzavirus emerged in North America and rapidly disseminated worldwide. First identified in Australia on May 12 2009, this virus was the dominant circulating influenza strain through the Australian winter; as of October 16, over 37 000 laboratory-confirmed cases of pandemic A(H1N1)2009 influenzavirus infection had been recorded, resulting in nearly 5 000 hospitalizations and 186 deaths [18]. Early reports suggested that pandemic A(H1N1)2009 influenzavirus infection differed from seasonal influenzavirus infection in that it spared older individuals, whilst moderate/severe infection was relatively common in younger patients[19], [20]. This pattern was also seen in Australia and was associated with significant utilization of ICU resources [21].

This report describes the features of severe pneumonia caused by pandemic A(H1N1)2009 influenzavirus/cMRSA co-infection in Western Australia, where cMRSA has been endemic for >15 years [17].

## Materials and Methods

### Case Identification

Patients hospitalized with laboratory–confirmed pandemic A(H1N1)2009 influenzavirus infection between July and September 2009 were prospectively identified at two adult teaching hospitals in Perth, Western Australia (Royal Perth Hospital [850 beds] and Sir Charles Gairdner Hospital [650 beds]). From this data, patients with community-acquired pneumonia (CAP) caused by co-infection with pandemic A(H1N1)2009 influenzavirus and MRSA were identified. In addition, cases of pandemic A(H1N1)2009 influenzavirus/MRSA co-infection identified at post-mortem examination performed at the State Forensic Pathology service during the same period were identified. Clinical information was obtained by chart review and for deceased patients, from general practitioners and/or paramedical staff. As this study was considered to be audit or ‘low risk’ research activity according to institutional and National Health and Medical Research Council criteria [22], [23], formal ethics committee approval and informed consent from the patients/next of kin were not required.

### Definitions

Community-acquired pneumonia was diagnosed if the patient had not been hospitalized within the preceding 3 months and had either a) symptoms and/or signs of lower respiratory tract infection together with pulmonary consolidation or infiltrates on imaging, or b) both macroscopic and microscopic evidence of pneumonia on post-mortem examination. Pandemic A(H1N1)2009 influenzavirus infection was diagnosed if the patient had symptoms or signs of an ILI and returned a positive result for pandemic A(H1N1)2009 influenzavirus RNA by polymerase chain reaction (PCR) on a respiratory tract specimen. Community-onset MRSA (cMRSA) infection was diagnosed if the patient returned a positive culture result for MRSA from blood cultures and/or from a lower respiratory tract specimen <48h following admission; patients from long-term care facilities were also included.

### MRSA Identification and Characterization

MRSA was isolated and identified from clinical specimens using routine laboratory methods. Cefoxitin, penicillin, clindamycin, erythromycin, tetracycline, trimethoprim, ciprofloxacin, gentamicin, rifampin, fusidic acid and mupirocin susceptibility testing was performed by disk diffusion according to Clinical and Laboratory Standards Institute (CLSI) recommendations [24]. CLSI interpretive criteria [25] were used for all antimicrobials except fusidic acid [26] and mupirocin [27]. Cefoxitin resistance was confirmed by the detection of the *mecA* gene by PCR [28]. Susceptibility testing for vancomycin and linezolid was performed by Etest (AB Biodisk, Solna, Sweden) and results interpreted according to CLSI breakpoints [29]. Pulsed-field gel electrophoresis (PFGE) of chromosomal DNA following *Sma*1 enzyme restriction was performed as previously described [30] using the CHEF DR III System (Bio-Rad Laboratories Pty Ltd). Patterns were examined visually, scanned with a Quantity One device (Bio-Rad Laboratories Pty Ltd), digitally analyzed using FPQuest (Bio-Rad Laboratories), and grouped according to the criteria of Tenover *et al.* [31]. Chromosomal DNA for multilocus sequence typing (MLST) and *spa* typing was prepared using the DNeasy Tissue kit (Qiagen Pty Ltd). MLST was performed as previously described [32]; sequences were compared with those on the MLST website to assign a sequence type (ST) (http://saureus.mlst.net/). *Spa* typing was performed as previously described [33] and *spa* types assigned as per standard methodology (http://spa.ridom.de/). The SCC*mec* element was typed by multiplex PCR [34]. Detection of the PVL genes (*luk*S-PV and *luk*F-PV) was performed by PCR as previously described [35]. Finally, gene profiling using a *S. aureus* specific DNA microarray was performed using the CLONDIAG platform; protocols, data interpretation and evaluation procedures for the oligonucleotide array hybridizations were performed as previously described [36], [37].

### Detection of Pandemic A(H1N1)2009 Influenzavirus

Testing of respiratory tract specimens for influenzavirus was performed on request; in addition, one centre routinely tested lower respiratory tract specimens from hospitalized patients that were submitted for bacterial culture during July–September 2009. Upper respiratory tract specimens included nose and throat swabs collected using either plastic-shafted Dacron swabs placed into viral transport medium (VTM) or cotton-tipped wire swabs that were vortexed in VTM in the laboratory. Lower respiratory tract specimens included expectorated sputum, sputum aspirated via an endotracheal tube, bronchoalveolar lavage fluid and lung sections obtained at post-mortem examination.

Three duplex real-time reverse transcriptase-PCR assays were run on each specimen to detect matrix gene targets specific for influenza A and influenza B, hemaaglutinin gene targets specific for influenza A subtypes H1 (seasonal), H1 (pandemic) and H3, and MS2 RNA coliphage (MS2) to monitor the efficiency of the assay [S. Williams, G. Chidlow, D.W. Smith, manuscript submitted]. Briefly, nucleic acid was extracted from 200 µL sample volume, and a standardized amount of MS2 was added to the specimen to monitor sample extraction efficiency, the removal of reverse transcription and PCR inhibitors and the cDNA production process [38]. Primers and probes [listed in Table S1] were designed using Primer Express software (Applied Biosystems, USA) with the exception of those for the influenza A matrix gene [39]; primers and probes to detect regions of the haemagglutinin gene that differentiated the pandemic A(H1N1)2009 from seasonal strains were designed based on sequence information obtained from the Global Initiative on Sharing Avian Influenza data (GISAID) (http://platform.gisaid.org/). DNA amplification was performed in real-time thermocyclers (RotorgeneQ, Qiagen, Germany); reactions with cycling threshold values <37 were reported as reactive. Negative samples that showed inhibition in the MS2 PCR were diluted 1∶5 and repeated. An evaluation of the pandemic A(H1N1)2009 PCR showed that it detected approximately 100 TCID_50_/mL at a 95% confidence level, with a diagnostic sensitivity of 98.8% and a specificity of 100% [S. Williams, G. Chidlow , D.W.Smith, manuscript submitted].

### Examination of Lung Specimens from Fatal Cases

When macroscopically abnormal lungs were observed at post-mortem examinations, samples of lung tissue were obtained and submitted for histopathological examination, bacterial and fungal microscopy and culture, and detection of respiratory tract pathogens by PCR.

## Results

252 patients with pandemic A(H1N1)2009 influenzavirus infection were admitted to the two participating hospitals during the 3-month period of study (149 to Royal Perth Hospital and and 103 to Sir Charels Gairdner Hospital). From these cases, 3 cases of co-infection with pandemic A(H1N1)2009 influenzavirus/cMRSA co-infection were identified (1.2% of all admissions with pandemic A(H1N1)2009 influenzavirus). In addition, 2 cases of pandemic A(H1N1)2009 influenzavirus/cMRSA co-infection were identified at post-mortem examination during the same time period.

### Clinical Details

The clinical characteristics of the cases of cMRSA/pandemic A(H1N1)2009 influenzavirus co-infection are summarized in table 1. There were 3 female and 2 males, aged between 34 and 79 years. Three cases (patients 1–3) were diagnosed following hospital presentation and two (patients 4 and 5), who died at home, were diagnosed following post-mortem examination. Two patients lived at the same long-term care facility, whilst the other patients lived independently in the community. Four of the 5 patients had conditions that may have increased their risk of pneumonia, including quadriplegia (two patients) asthma (one patient), cirrhosis (one patient) and diabetes mellitus (one patient). Two of the 5 cases (patients 3 and 4) had known MRSA infection/colonization prior to the onset of their illness (with the same cMRSA clone that subsequently caused their co-infection).

**Table 1:** Characteristics of patients with community-acquired pneumonia due to pandemic A(H1N1)2009 influenzavirus/cMRSA co-infection.

patient	symptoms	symptom duration before presentation/death	PSI	MRSA results (specimen, day of admission)	pandemic A(H1N1)2009 results [specimen, (day of admission)]	empiric therapy	definitive therapy (duration)	ICU admission	length of hospital stay (d)	outcome
1	cough, rigors, fever	3d	73 (class II)	blood (d1)sputum (d3,8)BAL (d8)pleural fluid (d12)	nose/throat (d1) −vesputum (d8),(d16) +veBAL (d8) +veinfluenza A CFT titre 160 (d1) and >320 (d8)	OTVCFTAZI	OTV 75mg bid (10d)VAN + CLI (7d)LIN (39d)CLI (28d)	Y	46	survived
2	cough with sputum, dyspnoea	3d	106 (class IV)	blood (d1)sputum (d1)	nose/throat (d1) −vesputum (d1) +ve	OTVAMXAZI	OTV 75mg bid (5d)VAN (14d)	N	9	survived
	**symptoms**	**symptom duration before presentation/death**	**PSI**	**MRSA results (specimen, day of admission)**	**pandemic A(H1N1)2009 results [specimen, (day of admission)]**	**empiric therapy**	**definitive therapy (duration)**	**ICU admission**	**length of hospital stay (d)**	**outcome**
3	cough with sputum, dyspnoea, pleuritic chest pain fever	5d	113 (class IV)	blood (d1)sputum (d1)	nose/throat (d1) +ve, (d7) −ve	AMX-CLA (PO) + CIP (PO) (pre-hospital)OTVCFTAZI	OTV 150mg bid(10d)VAN + LIN (7d)LIN (7d)	N	12	survived
4	confusion, dyspnoea, fever, myalgias	1d	-	right lower lobe (also cultured *E. coli* and methicillin susceptible *S. aureus*)	right lower lobe	-	-	-	-	died prior to hospital presentation
5	R abdominal pain	unknown	-	right middle lobe	right middle lobe	-	-	-	-	died prior to hospital presentation

**Note**:; PSI = Pneumonia Severity Index; BAL = bronchoalveolar lavage; CFT = complement fixation titre; OTV = oseltamivir; CFT = ceftriaxone; AZI = azithromycin; AMX = amoxicillin; AMX-CLA = amoxicillin-clavulanic acid; PO = per oral; CIP = ciprofloxacin; VAN = vancomycin; CLI = clindamycin; LIN = linezolid; ICU = intensive care unit.

**Figure 1:**
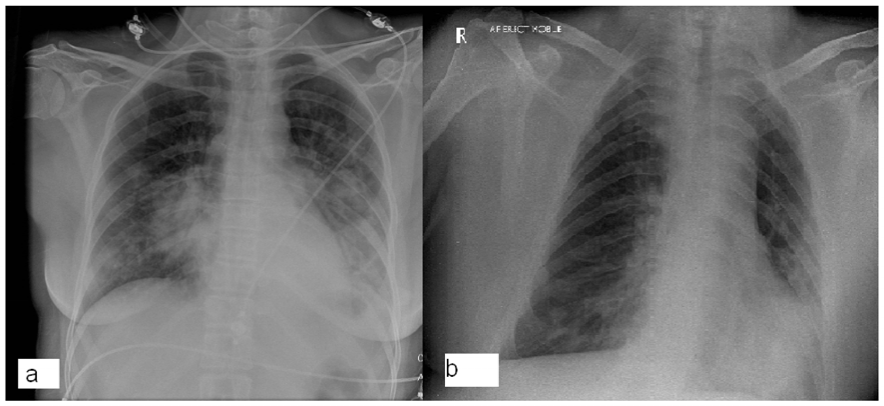
Chest radiography in pandemic A(H1N1)2009/cMRSA co-infection. (a) patient 1, (b) patient 3.

**Figure 2:**
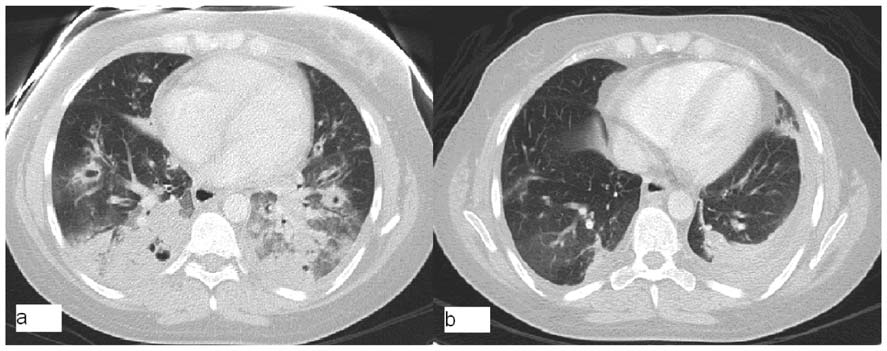
Chest computerized tomography scan in pandemic A(H1N1)2009/cMRSA co-infection. Patient 1, (a) 8 days and (b) 27 days following admission.

The three patients that presented to hospital required admission, with hospital length of stay ranging from 9–46 days. All three patients had ILI prior to presentation (duration from 3–5 days), and all had clinical and imaging features of pneumonia at presentation (see figures 1 and 2 for representative imaging) with Pneumonia Severity Index (PSI) score ranging from 74 (class II) to 113 (class IV). One patient (patient 1, PSI score = 73) required intensive care unit admission for mechanical ventilation on day 8 of admission, and another patient (patient 3) received bi-level positive airways pressure (BiPAP) ventilation on a respiratory ward.

Patients 4 and 5 died at home not having presented for medical assessment. Patient 4 was noted to have an ILI when visited by domiciliary nurses three days prior to death; when contacted by a primary care practitioner on the day of death, the patient was confused and dyspnoiec, and subsequently suffered cardiorespiratory arrest when paramedics attended shortly afterwards. Post-mortem examination findings included acute pulmonary oedema, right lower lobe consolidation, and moderate coronary artery disease; the cause of death was recorded as acute pneumonia. Patient 5 had complained to a relative of being generally unwell with right-sided abdominal pain 3 days before they were found deceased; post-mortem examination demonstrated right middle lobe pneumonia, with previously undiagnosed disseminated small cell carcinoma with hepatic metastases, and moderate to severe coronary artery disease with cardiomegaly; the cause of death was recorded as acute pneumonia.

### Microbiology Results

The three patients who presented to hospital had pandemic A(H1N1)2009 influenzavirus detected by PCR in upper and/or lower respiratory tract specimens. Two patients (patient 1 and patient 2) had nose and throat swabs taken at admission that tested negative for pandemic A(H1N1)2009 influenzavirus; however both patients had positive results on lower respiratory tract specimens (patient 1 from sputum aspirated from the endotracheal tube on day 8 of admission, and patient 2 on expectorated sputum obtained day 1 of admission). Two patients (patients 1 and 3) had surveillance specimens obtained after commencement of therapy; patients 1 remained positive for pandemic A(H1N1)2009 influenzavirus RNA by PCR on endotracheal sputum on day 16 of admission (following two 5-day courses of oseltamivir), whereas patient 3 was negative on repeat nose/throat swab on day 7 of admission (whilst still receiving oseltamivir). The two cases identified at post-mortem examination had pandemic A(H1N1)2009 influenzavirus RNA detected on tissue samples from macroscopically abnormal lung; no other viral pathogens were identified. In addition, patient 1 had a >4 fold rise in influenza A antibody titre on specimens collected on the day of admission and day 8 of admission.

The three patients who presented to hospital all had MRSA cultured from blood and from expectorated sputum specimens collected within 12h of admission. Two of these patients (patients 2 and 3) had negative surveillance blood cultures 72–96h after admission; the remaining patient (patient 1) did not have surveillance blood cultures performed until day 26 of admission (which were negative), but was culture-positive for MRSA on a pleural fluid specimen obtained 11 days after the commencement of MRSA therapy. The two post-mortem cases had Gram-positive cocci resembling staphylococci present on Gram's stain of tissue submitted to the microbiology laboratory, and cultured MRSA from lung specimens; one patient also cultured methicillin-susceptible *S. aureus* and *E. coli* but these organisms were present in relatively low numbers on culture.

### Histopathology

**Figure 3:**
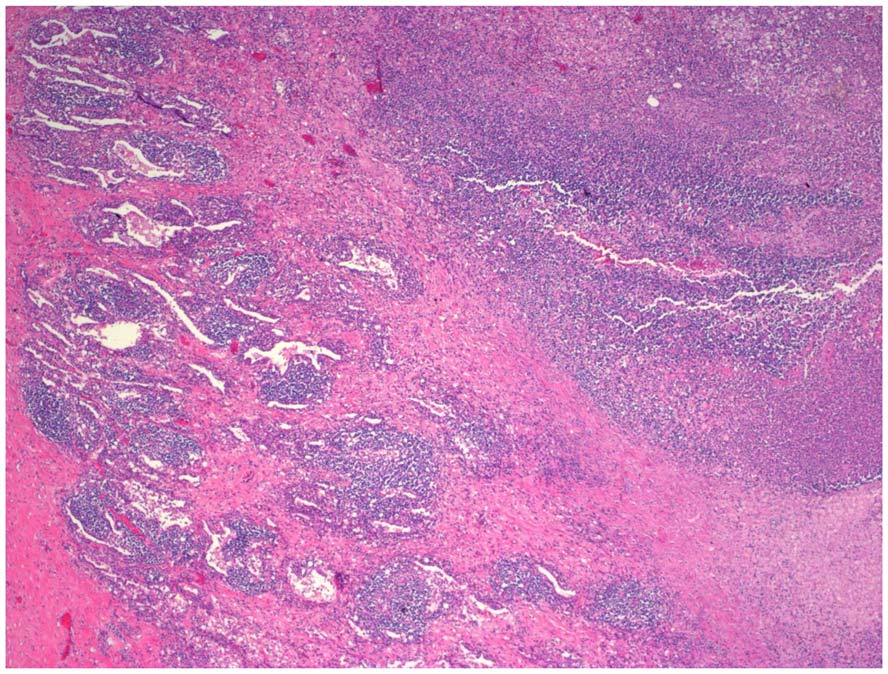
Histopathology of pandemic A(H1N1)/cMRSA co-infection. Patient 5, right lower lobe lung, demonstrating areas of necrotizing pneumonia (haematoxylin and eosin, ×40).

**Figure 4:**
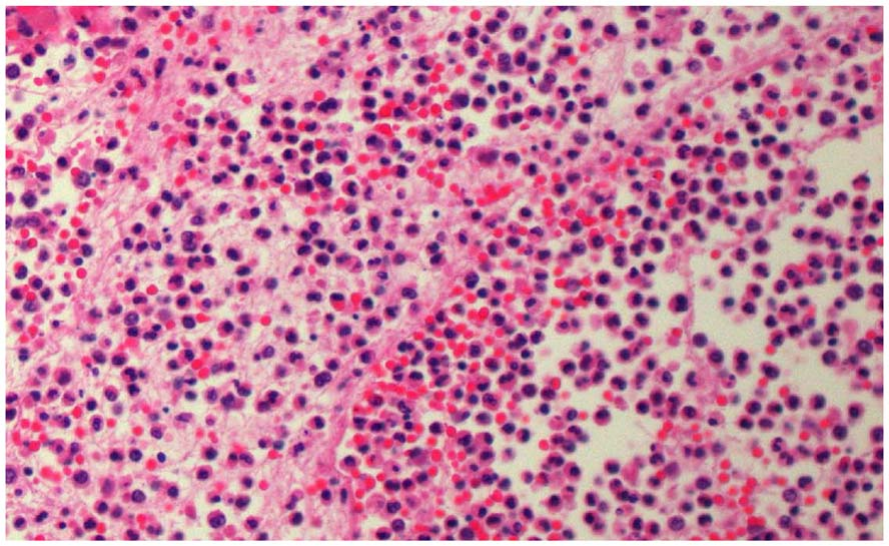
Histopathology of pandemic A(H1N1)/cMRSA co-infection. Patient 4, right lower lobe lung, demonstrating acute pneumonia (haematoxylin and eosin, ×200).

Histopathological examination of lung obtained from patients 4 and 5 demonstrated features of acute and/or necrotizing pneumonia (figures 3 and 4).

### Treatment of Pandemic A(H1N1)2009 Infection

The three patients who presented to hospital received empiric therapy for pandemic A(H1N1)2009 influenzavirus infection with oseltamivir, at doses of 75mg bid or 150mg bid, for durations of between 5 and 10 days. Patient 1 initially received 5 days of oseltamivir, which was ceased when nose/throat swabs returned negative results; following receipt of positive results for pandemic A(H1N1)2009 influenzavirus on sputum on day 8 of admission, oseltamivir was re-introduced at the same dose and continued for another 5 days.

### Treatment of cMRSA Infection

None of the patients received empiric antimicrobial therapy that would be considered appropriate for bacteraemic cMRSA pneumonia (including patient 3, who had a past history of MRSA infection). All 3 patients received vancomycin following receipt of positive blood culture results or sputum microscopy with gram-positive cocci resembling staphylococci in Gram's stain (empiric therapy with vancomycin in combination with an antistaphylococcal β-lactam such as flucloxacillin is standard therapy for suspected community-acquired *S. aureus* sepsis in our region). Duration of MRSA therapy ranged from 14 to 76 days; patient 1 received combination therapy with vancomycin and clindamycin for 7 days, followed by linezolid (IV then PO) for 39 days, followed by oral clindamycin on discharge to complete a further 4 weeks of therapy; patient 2 received vancomycin monotherapy for 14 days (5 as an outpatient), and patient 3 received 7 days of vancomycin and oral linezolid; vancomycin was ceased after 7 days because of neutropenia and treatment was continued with oral linezolid to complete a total of 14 days of therapy.

### Outcomes

All patients who survived until hospital presentation were discharged without permanent sequelae.

### MRSA Typing Results

**Figure 5:**
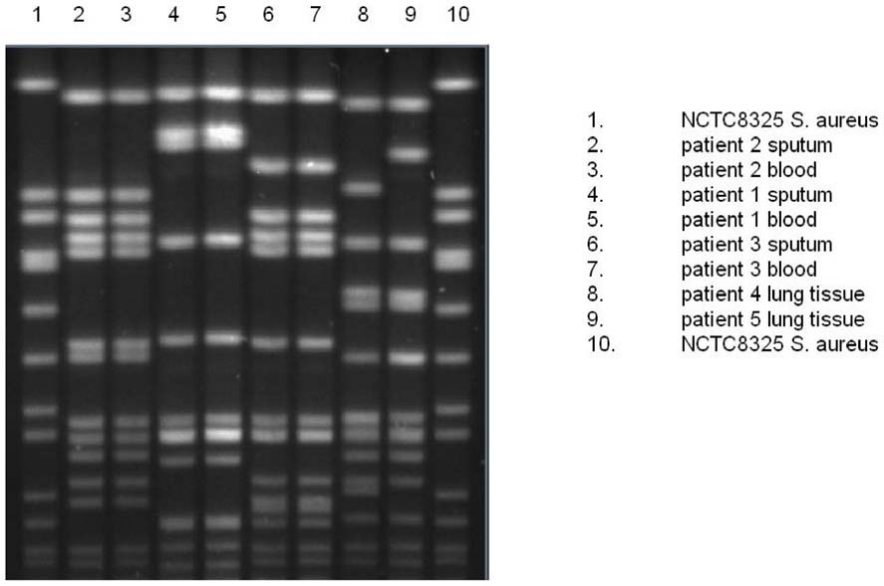
Pulsed-field gel electrophoresis of MRSA isolates (*Sma*1 macrorestriction).

Three different cMRSA clones were identified in 5 patients (Table 2 and Figure 5). These cMRSA clones (ST1-MRSA-IV, ST93-MRSA-IV and ST78-MRSA-IV) are all known to circulate in the Western Australian community [17]. Only the isolate from patient 1 (ST93-MRSA-IV) contained the PVL toxin genes *lukS*-PV/*lukF*-PV; this patient had a severe and protracted clinical course consistent with necrotizing pneumonitis, which was supported by imaging findings (figure 3). Patients 2 and 3, who resided at the same long-term care facility, had MRSA isolates that were indistinguishable by MLST (ST1-MRSA-IV) but had different PFGE patterns (3-band difference) and different *spa* types. Patients 4 and 5 had ST78-MRSA-IV isolated from lung tissue (indistinguishable PFGE, spa type t186) however there was no known epidemiological connection between these two cases.

**Table 2:** MRSA typing results.

patient	source	antibiogram	*luk*S-PV/*luk*F PV PCR	PFGE designation	MLST sequence	MLST sequence type (clonal complex)	*spa* type	SCC*mec* type	*agr* type
1	sputum	S	+	Qld CA-MRSA	6-64-44-2-43-55-51	93 (singleton)	t202	IVa	III
1	blood	S	+	Qld CA-MRSA	6-64-44-2-43-55-51	93 (singleton)	t202	IVa	III
2	sputum	S	−	WA MRSA-1	1-1-1-1-1-1-1	1 (1)	t177	IVa	III
2	blood	S	−	WA MRSA-1	1-1-1-1-1-1-1	1 (1)	t177	IVa	III
3	sputum	S	−	WA MRSA-1	1-1-1-1-1-1-1	1 (1)	t127	IVa	III
3	blood	S	−	WA MRSA-1	1-1-1-1-1-1-1	1 (1)	t127	IVa	III
4	lung	Ery^R^, IRC	−	WA MRSA-2	22-1-14-23-12-53-31	78 (88)	t186	IVa	III
5	lung	Ery^R^, IRC	−	WA MRSA-2	22-1-14-23-12-53-31	78 (88)	t186	IVa	III

**Note:** S = isolate tested susceptible to all non-beta lactam agents tested; Ery^R^ = erythromycin resistant; IRC = inducible resistance to clindamycin;*luk*S-PV/*luk*F-PV = genes encoding Panton-Valentine Leukocidin; PFGE = pulsed-field gel electrophoresis; MLST = multilocus sequence typing; *spa* = staphylococcal protein A gene; *agr* = accessory gene regulator.

Results of DNA microarray experiments on MRSA isolated from the cases were 100% concordant with PCR results for *lukS*-PV/*lukF*-PV and sequencing results for MLST. All of the MRSA isolates were *agr* group III; none contained the arginine catabolic mobile element (ACME) locus. All isolates contained the genes for delta-haemolysin (*hld*), putative haemolysin III *(hIII)*, staphylokinase *(Sak)*, *and* staphylococcal complement inhibitor *(Scn)*; genes for alpha-haemolysin (*hla)*, beta-haemolysin *(hlb)*, gamma-haemolysin (*lukF* and *lukS*), putative haemolysins *hlIII* and leukocidin genes *lukD/E* were present in the four *lukS*-PV/*lukF*-PV-negative isolates. Enterotoxin genes were detected in three isolates (*entA*, *entH*, *entK* and *entQ* in isolates from patient 2, *entA* and *entH* in isolates from patient 3 and *entC* and *entL* in isolates from patient 5). Genes encoding the following microbial surface components recognizing adhesive matrix molecules (MSCRAMMS) were detected in all isolates: bone sialoprotein-binding protein (*bbp*), clumping factors A *(clfA)* and B *(clfB)*, cell-wall associated fibronectin binding protein *(ebh)*, cell surface elastin-binging protein *(ebpS)*, enolase *(eno)*, fibrinogen-binding protein *(fib)*, fibronectin binding proteins *A (fnbA)* and B *(fnbB)*, Serine-aspartate rich fibrinogen binding proteins C*(sdrC)* and D*(sdrD)*, and von Willebrand Factor binding protein *(vwb)*. Complete results of the DNA microarray experiments are presented in table S2, and the raw data is available from the authors on request.

## Discussion

Initial descriptions of severe pandemic A(H1N1)2009 influenzavirus infection reported from the United States were characteristic of a viral pneumonitis, with bacterial co-infection or sequential infection being relatively uncommon [19], [20]. In particular, MRSA co-infection occurred in only 1/272 patients hospitalized with pandemic A(H1N1)2009 influenzavirus infection reported to the US Centers for Disease Control between April and June 2009 [20]. As the pandemic has progressed, evidence has emerged that bacterial infection occurs in some patients, although the exact incidence and contribution to morbidity and mortality has yet to be determined. Post-mortem lung specimens from 77 cases of fatal A(H1N1)2009 influenzavirus infection demonstrated histopathological, immunohistochemical and/or molecular evidence of concurrent bacterial infection in 22 (29%); 7 had *S. aureus* identified, 5 of which were MRSA [40]. As minimal clinical information was provided in this report, it is not known whether appropriate empiric antimicrobial therapy was given to these patients, although 4 had received antibacterial and antiviral therapy. In addition, a recent report described a case of fatal cMRSA/pandemic A(H1N1)2009 influenzavirus infection in a previously well 42-year-old male who died within 48h of admission [41]; this patient did not receive antiviral therapy, but did receive vancomycin and clindamycin.

The clinical features of pandemic A(H1N1)2009 influenzavirus/cMRSA co-infection appear similar to those described previously with seasonal influenzavirus/cMRSA co-infection [7]–[12]. All patients described an ILI prior to presentation/death, and the three patients who presented to hospital had moderately severe CAP and MRSA bacteremia. Pandemic A(H1N1)2009 influenzavirus RNA was not detected on nose/throat swabs in 2 of these three cases, but was detected on lower respiratory tract samples. We therefore recommend that in addition to upper respiratory tract specimens, lower respiratory tract specimens should be tested for pandemic A(H1N1)2009 influenzavirus if the patient has pneumonia.

The three patients with pandemic A(H1N1)2009 influenzavirus/cMRSA co-infection who were admitted to hospital received empiric therapy with oseltamivir, however none received empiric therapy considered appropriate for invasive cMRSA infection. This was in spite of the fact that two of these patients were known to be colonized with MRSA prior to developing pneumonia. The small number of patients in this report makes it impossible to draw any conclusions regarding the impact of appropriate empiric therapy for either pandemic A(H1N1)2009 influenzavirus or for cMRSA.

Only one of the 5 cases of pandemic A(H1N1) influenzavirus/cMRSA co-infection had infection with a cMRSA clone containing the genes encoding PVL toxin (ST93-MRSA-IV). This is consistent with recent data from our region suggesting that the PVL is not essential for the development of severe cMRSA CAP [42]. The patient with PVL-containing MRSA infection did, however, have a severe and protracted pulmonary infection consistent with necrotizing pneumonia that progressed despite early therapy with the protein synthesis inhibitor clindamycin, similar to the previous case report of pandemic A(H1N1) influenzavirus co-infection which was caused by another PVL-containing cMRSA clone (ST30-MRSA-IV)[41].

This study has several limitations. Firstly, it was performed in only two adult tertiary hospitals in an isolated Australian city, so the results may not be generalizable to the pediatric population or to regions with differing cMRSA prevalence. Secondly, patients with mild-to-moderate CAP (who often do not have microbiological tests performed) were not represented, therefore the data may overestimate the severity of co-infection. Finally, as we did not perform immunohistochemical staining, we could not conclusively demonstrate that MRSA was present in the two post-mortem lung specimens, however, histopathological findings were suggestive of an acute bacterial pneumonia, and cMRSA was the dominant organism isolated on culture.

This report emphasizes the importance of bacterial co-infection in pandemic A(H1N1)2009 influenzavirus infection. Clinicians should be alert to the possibility of cMRSA co-infection in patients with suspected influenza pneumonitis, should ensure that appropriate lower respiratory tract specimens are obtained for the detection of influenzavirus and MRSA, and should consider instituting empiric antibacterial therapy with activity against MRSA in regions where cMRSA is prevalent.

## Supporting Information

Table S1 Primers and probes included in the duplex real-time RT-PCR assays.
(0.05 MB DOC)

Table S2 Complete results of DNA microarray experiments on MRSA isolates from patients with community-acquired pneumonia due to pandemic A(H1N1)2009/cMRSA co-infection.
(0.42 MB DOC)
