# Supplementary material for: Community-Acquired Pneumonia Due to Pandemic A(H1N1)2009 Influenzavirus and Methicillin Resistant Staphylococcus aureus Co-Infection
Source: PLoS One. 2010 Jan 14;5(1):e8705. doi: 10.1371/journal.pone.0008705 (PMC2806836; doi:10.1371/journal.pone.0008705)
Supplement: Table S1 — Primers and probes included in the duplex real-time RT-PCR assays. (0.05 MB DOC) [file pone.0008705.s001.doc]

**Table S1. Primers and probes included in the duplex real-time RT-PCR assays.**

| Mix | Target | Primer/Probe^a^ | Sequence 5’-3’ | Gene Target | Product |
| --- | --- | --- | --- | --- | --- |
| 1 | Influenza A Matrix [39] | FLUA-MAT-F  FLUA-MAT-R  FA-MAT-PR | CTTCTAACCGAGGTCGAAACGTA  GGTGACAGGATTGGTCTTGTCTTTA  CALO-TCAGGCCCCCTCAAAGCCGAG-BHQ1 | Matrix protein | 155bp |
| 1 | Influenza B | FBMAT-24  FBMAT-98  FBMAT-51 | TGCCTACCTGCTTTMMYTRACA  CCRAACCAACARTGTAATTTTTCTG  6FAM-TGCTTTGCCTTCTCCA-MGBNFQ | Matrix protein | 75bp |
| 2 | Influenza A Pandemic (H1N1) 2009 | SWHA-440  SWHA-545  SWHA-465 | AAGGTGTAACGGCAGCATGTC  TAGGATTTGCTGAGCTTTGGGTAT  6FAM-AGAAGCTTTTTGCTCCAGCA-MGBNFQ | Haemagglutinin | 106bp |
| 2 | MS-2 RNA coliphage | MS2-105  MS2-170  MS2-127 | GTCGACAATGGCGGAACTG  TTCAGCGACCCCGTTAGC  CALO-ACGTGACTGTCGCCCCAAGCAACTT-BHQ1 | Coat protein | 66bp |
| 3 | Influenza A H1 | H1N1 HA-435  H1N1 HA-491  H1HA-454  H1HA-454mod | AAGCTCATGGCCCAACCA  CCATTATGGGAGCATGATGCT  VIC-ATACTCCGGTCACGGT-MGBNFQ  VIC-ACACTCCGGTTACGGT-MGBNFQ | Haemagglutinin | 57bp |
| 3 | Influenza A H3 | H3N2 HA-857  H3N2 HA-928  H3HA-889 | ACGAAGTGGGAAAAGCTCAATAAT  GGAGTGATGCATTCAGAATTGC  6FAM-ATGCACCCATTGGC-MGBNFQ | Haemagglutinin | 72bp |

^a^Forward primer listed first
