# Supplementary material for: Community-Acquired Pneumonia Due to Pandemic A(H1N1)2009 Influenzavirus and Methicillin Resistant Staphylococcus aureus Co-Infection
Source: PLoS One. 2010 Jan 14;5(1):e8705. doi: 10.1371/journal.pone.0008705 (PMC2806836; doi:10.1371/journal.pone.0008705)
Supplement: Table S2 — Complete results of DNA microarray experiments on MRSA isolates from patients with community-acquired pneumonia due to pandemic A(H1N1)2009/cMRSA co-infection. (0.42 MB DOC) [file pone.0008705.s002.doc]

**Table S2. Complete results of DNA microarray experiments on MRSA isolates from patients with community-acquired pneumonia due to pandemic A(H1N1)2009/cMRSA co-infection.**

| **Gene/Probe** | **Explanation** | **Patient #/**  **site** |  |  |  |  |  |  |  |
| --- | --- | --- | --- | --- | --- | --- | --- | --- | --- |
|  |  | **1** | **1** | **5** | **2** | **2** | **3** | **3** | **4** |
|  |  | **blood** | **sputum** | **lung** | **sputum** | **blood** | **sputum** | **blood** | **lung** |
| **Species Markers/Regulatory Genes** |  |  |  |  |  |  |  |  |  |
| 23S-rRNA | 23S-rRNA Gene | **POS** | **POS** | **POS** | **POS** | **POS** | **POS** | **POS** | **POS** |
| gapA | Glyceraldehyde 3-phosphate Dehydrogenase, locus 1 | **POS** | **POS** | **POS** | **POS** | **POS** | **POS** | **POS** | **POS** |
| katA | Catalase | **POS** | **POS** | **POS** | **POS** | **POS** | **POS** | **POS** | **POS** |
| coA | Coagulase | **AMB** | **POS** | **POS** | **POS** | **POS** | **POS** | **POS** | **POS** |
| Protein A | Staphylococcus Protein A | **POS** | **POS** | **POS** | **POS** | **POS** | **POS** | **POS** | **POS** |
| Sbi | IgG-binding protein | **POS** | **POS** | **POS** | **POS** | **POS** | **POS** | **POS** | **POS** |
| Nuc | Thermostable Nuclease (DNAse) | **POS** | **POS** | **POS** | **POS** | **POS** | **POS** | **POS** | **POS** |
| fnbA | Fibronectin-binding Protein A | **POS** | **POS** | **POS** | **POS** | **POS** | **POS** | **POS** | **POS** |
| vraS | vraS Sensor Protein | **POS** | **POS** | **POS** | **POS** | **POS** | **POS** | **POS** | **POS** |
| sarA | Staphylococcal Accessory Regulator A | **POS** | **POS** | **POS** | **POS** | **POS** | **POS** | **POS** | **POS** |
| Eno | Enolase, Phosphopyruvate Hydratase | **POS** | **POS** | **POS** | **POS** | **POS** | **POS** | **POS** | **POS** |
| saeS | Histidine Protein Kinase (sae Locus) | **POS** | **POS** | **POS** | **POS** | **POS** | **POS** | **POS** | **POS** |
| **Resistance Genotype** |  |  |  |  |  |  |  |  |  |
| mecA | Penicillin binding protein 2, betalactam resistance defining MRSA | **POS** | **POS** | **POS** | **POS** | **POS** | **POS** | **POS** | **POS** |
| blaZ | Beta-lactamase | **POS** | **POS** | **POS** | **POS** | **POS** | **POS** | **POS** | **POS** |
| blaI | Beta lactamase repressor (inhibitor) | **POS** | **POS** | **POS** | **POS** | **POS** | **POS** | **POS** | **POS** |
| blaR | beta-lactamase regulatory protein | **POS** | **POS** | **POS** | **POS** | **POS** | **POS** | **POS** | **POS** |
| ermA | Macrolide, Lincosamide, Streptogramin | NEG | NEG | NEG | NEG | NEG | NEG | NEG | **POS** |
| ermB | Macrolide, Lincosamide, Streptogramin | NEG | NEG | NEG | NEG | NEG | NEG | NEG | NEG |
| ermC | Macrolide, Lincosamide, Streptogramin | NEG | NEG | NEG | NEG | NEG | NEG | NEG | NEG |
| linA | Lincosamides | NEG | NEG | NEG | NEG | NEG | NEG | NEG | NEG |
| msrA | Macrolide | NEG | NEG | NEG | NEG | NEG | NEG | NEG | NEG |
| mefA | Macrolide | NEG | NEG | NEG | NEG | NEG | NEG | NEG | NEG |
| mpbBM | Macrolide | NEG | NEG | NEG | NEG | NEG | NEG | NEG | NEG |
| vatA | Streptogramin | NEG | NEG | NEG | NEG | NEG | NEG | NEG | NEG |
| vatB | Streptogramin | NEG | NEG | NEG | NEG | NEG | NEG | NEG | NEG |
| Vga | Streptogramin | NEG | NEG | NEG | NEG | NEG | NEG | NEG | NEG |
| vgaA | Streptogramin | NEG | NEG | NEG | NEG | NEG | NEG | NEG | NEG |
| Vgb | Streptogramin | NEG | NEG | NEG | NEG | NEG | NEG | NEG | NEG |
| aacA-aphD | Aminoglycoside (Gentamicin, Tobramycin) | NEG | NEG | NEG | NEG | NEG | NEG | NEG | NEG |
| aadD | Aminoglycoside (Gentamicin, Neomycin) | NEG | NEG | NEG | NEG | NEG | NEG | NEG | NEG |
| aphA | Aminoglycoside (Kanamycin, Tobramycin) | NEG | NEG | NEG | NEG | NEG | NEG | NEG | NEG |
| Sat | Streptothricin | NEG | NEG | NEG | NEG | NEG | NEG | NEG | NEG |
| dfrA | Trimethoprim | NEG | NEG | NEG | NEG | NEG | NEG | NEG | NEG |
| Far | Fusidic Acid | NEG | NEG | NEG | NEG | NEG | NEG | NEG | NEG |
| Q6GD50 | Putative Fusidic Acid Resistance Protein | NEG | NEG | NEG | NEG | NEG | NEG | NEG | NEG |
| mupR | Mupirocin | NEG | NEG | NEG | NEG | NEG | NEG | NEG | NEG |
| tetK | Tetracycline | NEG | NEG | NEG | NEG | NEG | NEG | NEG | NEG |
| tetM | Tetracycline | NEG | NEG | NEG | NEG | NEG | NEG | NEG | NEG |
| tetEfflux | Tetracycline Efflux Protein (Putative Transport Protein) | **POS** | **POS** | **POS** | **POS** | **POS** | **POS** | **POS** | **POS** |
| Cat | Chloramphenicol | NEG | NEG | NEG | NEG | NEG | NEG | NEG | NEG |
| fexA | Chloramphenicol | NEG | NEG | NEG | NEG | NEG | NEG | NEG | NEG |
| Cfr | Phenicols, Lincosamides, Oxazolidinones (Linezolid), Pleuromutilins, Streptogramin A | NEG | NEG | NEG | NEG | NEG | NEG | NEG | NEG |
| fosB | Putative Marker for Fosfomycin, Bleomycin | NEG | NEG | NEG | NEG | NEG | NEG | NEG | NEG |
| vanA | Vancomycin | NEG | NEG | NEG | NEG | NEG | NEG | NEG | NEG |
| vanB | Vancomycin | NEG | NEG | NEG | NEG | NEG | NEG | NEG | NEG |
| vanZ | Vancomycin | NEG | NEG | NEG | NEG | NEG | NEG | NEG | NEG |
| Mercury resistance locus | Mercury resistance operon | NEG | NEG | NEG | NEG | NEG | NEG | NEG | NEG |
| qacA | Non specific efflux pump | NEG | NEG | NEG | NEG | NEG | NEG | NEG | NEG |
| qacC | Non specific efflux pump | NEG | NEG | NEG | NEG | NEG | NEG | NEG | NEG |
| **Virulence Genotype** |  |  |  |  |  |  |  |  |  |
| tst-1 | Toxic Shock Syndrome | NEG | NEG | NEG | NEG | NEG | NEG | NEG | NEG |
| entA | Enterotoxin A | NEG | NEG | NEG | **AMB** | **POS** | **POS** | **POS** | NEG |
| entB | Enterotoxin B | NEG | NEG | NEG | NEG | NEG | NEG | NEG | NEG |
| entC | Enterotoxin C | NEG | NEG | **POS** | NEG | NEG | NEG | NEG | NEG |
| entD | Enterotoxin D | NEG | NEG | NEG | NEG | NEG | NEG | NEG | NEG |
| entE | Enterotoxin E | NEG | NEG | NEG | NEG | NEG | NEG | NEG | NEG |
| entG | Enterotoxin G | NEG | NEG | NEG | NEG | NEG | NEG | NEG | NEG |
| entH | Enterotoxin H | NEG | NEG | NEG | **POS** | **POS** | **POS** | **POS** | NEG |
| entI | Enterotoxin I | NEG | NEG | NEG | NEG | NEG | NEG | NEG | NEG |
| entJ | Enterotoxin J | NEG | NEG | NEG | NEG | NEG | NEG | NEG | NEG |
| entK | Enterotoxin K | NEG | NEG | NEG | **POS** | **POS** | NEG | NEG | NEG |
| entL | Enterotoxin L | NEG | NEG | **POS** | NEG | NEG | NEG | NEG | NEG |
| entM | Enterotoxin M | NEG | NEG | NEG | NEG | NEG | NEG | NEG | NEG |
| entN | Enterotoxin N | NEG | NEG | NEG | NEG | NEG | NEG | NEG | NEG |
| entO | Enterotoxin O | NEG | NEG | NEG | NEG | NEG | NEG | NEG | NEG |
| entQ | Enterotoxin Q | NEG | NEG | NEG | **POS** | **POS** | NEG | NEG | NEG |
| entR | Enterotoxin R | NEG | NEG | NEG | NEG | NEG | NEG | NEG | NEG |
| entU | Enterotoxin U | NEG | NEG | NEG | NEG | NEG | NEG | NEG | NEG |
| egc-cluster | Enterotoxins seg/sei/sem/sen/seo/seu | NEG | NEG | NEG | NEG | NEG | NEG | NEG | NEG |
| lukS-PV/  lukF-PV | Panton-Valentine Leukocidin | **AMB** | **POS** | NEG | NEG | NEG | NEG | NEG | NEG |
| lukF | Haemolysin Gamma Componet B | NEG | NEG | **POS** | **POS** | **POS** | **POS** | **POS** | **POS** |
| lukS | Haemolysin Gamma Componet C | NEG | NEG | **POS** | **POS** | **POS** | **POS** | **POS** | **POS** |
| hlgA | Haemolysin Gamma Componet A | NEG | NEG | **POS** | NEG | NEG | NEG | **POS** | NEG |
| lukD | Leukocidin D Component | NEG | NEG | **POS** | **POS** | **POS** | **POS** | **POS** | **POS** |
| lukE | Leukocidin E Component | NEG | NEG | **POS** | **POS** | **POS** | **POS** | **POS** | **POS** |
| lukX | Leukocidin/Haemolysin Toxin Family Protein | **AMB** | **POS** | **POS** | **POS** | **POS** | **POS** | **POS** | **POS** |
| lukY | Leukocidin/Haemolysin Toxin Family Protein | NEG | NEG | **POS** | **POS** | **POS** | **POS** | **POS** | **POS** |
| Hl | Hypothetical Protein similar to Haemolysin | NEG | NEG | **POS** | **POS** | **POS** | **POS** | **POS** | **POS** |
| Hla | Haemolysin Alpha (Alpha Toxin) | NEG | NEG | **POS** | **POS** | **POS** | **POS** | **POS** | **POS** |
| Hld | Haemolysin Delta (Amphiphylic Membrane Toxin) | **POS** | **POS** | **POS** | **POS** | **POS** | **POS** | **POS** | **POS** |
| hlIII | Putative Haemolysin III | **POS** | **POS** | **POS** | **POS** | **POS** | **POS** | **POS** | **POS** |
| Hlb | Haemolysin Beta (Phospholipase C) | NEG | **POS** | **POS** | **POS** | **POS** | **POS** | **POS** | **POS** |
| Untuncated hlb | Haemolysin Beta (Phospholipase C untuncated) | NEG | NEG | NEG | NEG | NEG | NEG | NEG | NEG |
| Sak | Staphylokinase | **POS** | **POS** | **POS** | **POS** | **POS** | **POS** | **POS** | **POS** |
| Chp (CHIPS) | Chemotaxis Inhibitory Protein | **POS** | **POS** | NEG | NEG | NEG | **POS** | **POS** | NEG |
| Scn | Staphylococcal Complement Inhibitor | **POS** | **POS** | **POS** | **POS** | **POS** | **POS** | **POS** | **POS** |
| etA | Exfoliative Toxin A | NEG | NEG | NEG | NEG | NEG | NEG | NEG | NEG |
| etB | Exfoliative Toxin B | NEG | NEG | NEG | NEG | NEG | NEG | NEG | NEG |
| etD | Exfoliative Toxin D | NEG | NEG | NEG | NEG | NEG | NEG | NEG | NEG |
| edinA | Epidermal Cell Differentiation Inhibitor A | NEG | NEG | NEG | NEG | NEG | NEG | NEG | NEG |
| edinB | Epidermal Cell Differentiation Inhibitor B | NEG | NEG | NEG | NEG | NEG | NEG | NEG | NEG |
| edinC | Epidermal Cell Differentiation Inhibitor C | NEG | NEG | NEG | NEG | NEG | NEG | NEG | NEG |
| Aur | Aureolysin | **POS** | **POS** | **POS** | **POS** | **POS** | **POS** | **POS** | **POS** |
| splA | Serine Protease A | **POS** | **POS** | **POS** | **POS** | **POS** | **POS** | **POS** | **POS** |
| splB | Serine Protease B | NEG | NEG | **POS** | **POS** | **POS** | **POS** | **POS** | **POS** |
| splE | Serine Protease E | NEG | **AMB** | NEG | **POS** | **POS** | **POS** | **POS** | NEG |
| sspA | Glutamyl Endopeptidase/V8-Protease | **POS** | **POS** | **POS** | **POS** | **POS** | **POS** | **POS** | **POS** |
| sspB | Staphopain B | **POS** | **POS** | **POS** | **POS** | **POS** | **POS** | **POS** | **POS** |
| sspP | Staphopain A | **POS** | **POS** | **POS** | **POS** | **POS** | **POS** | **POS** | **POS** |
| ACME-locus | Arginine Catabolic Mobile Element | NEG | NEG | NEG | NEG | NEG | NEG | NEG | NEG |
| arcA-SCC | Arginine deiminase | NEG | NEG | NEG | NEG | NEG | NEG | NEG | NEG |
| arcB-SCC | Ornithine transcarbamoylase | NEG | NEG | NEG | NEG | NEG | NEG | NEG | NEG |
| arcC-SCC | Carbamate kinase, locus 2 | NEG | NEG | NEG | NEG | NEG | NEG | NEG | NEG |
| arcD-SCC | Arginine/ornithine antiporter | NEG | NEG | NEG | NEG | NEG | NEG | NEG | NEG |
| **agr Typing** |  |  |  |  |  |  |  |  |  |
| agrI | Accessory Gene Regulator – Type 1 | NEG | NEG | NEG | NEG | NEG | NEG | NEG | NEG |
| agrII | Accessory Gene Regulator – Type 2 | NEG | NEG | NEG | NEG | NEG | NEG | NEG | NEG |
| agrIII | Accessory Gene Regulator – Type 3 | **POS** | **POS** | **POS** | **POS** | **POS** | **POS** | **POS** | **POS** |
| agrIV | Accessory Gene Regulator – Type 4 | NEG | NEG | NEG | NEG | NEG | NEG | NEG | NEG |
| **SCCmec Typing** |  |  |  |  |  |  |  |  |  |
| mecA | Methicillin, Oxacillin and all Beta-Lactams defining MRSA | **POS** | **POS** | **POS** | **POS** | **POS** | **POS** | **POS** | **POS** |
| mecR | Signal Transducer Protein mecR1 | NEG | NEG | NEG | NEG | NEG | NEG | NEG | NEG |
| mecR-truncated | Signal Transducer Protein mecR1-truncated | **POS** | **POS** | **POS** | **POS** | **POS** | **POS** | **POS** | **POS** |
| mecI | Methicillin-Resistance Regulatory Protein | NEG | NEG | NEG | NEG | NEG | NEG | NEG | NEG |
| ugpQ | Glycerophosphoryl-diester-Phosphodiesterasse | **POS** | **POS** | **POS** | **POS** | **POS** | **POS** | **POS** | **POS** |
| ccrA-1 | Cassette Chromosome Recombinase A, type 1 | NEG | NEG | NEG | NEG | NEG | NEG | NEG | NEG |
| ccrA-2 | Cassette Chromosome Recombinase A, type 2 | **POS** | **POS** | **POS** | **POS** | **POS** | **POS** | **POS** | **POS** |
| ccrA-3 | Cassette Chromosome Recombinase A, type 3 | NEG | NEG | NEG | NEG | NEG | NEG | NEG | NEG |
| ccrA-MRSAZH47 | Cassette Chromosome Recombinase A, type ZH47 | NEG | NEG | NEG | NEG | NEG | NEG | NEG | NEG |
| ccrA-4 | Cassette Chromosome Recombinase A, type 4 | NEG | NEG | NEG | NEG | NEG | NEG | NEG | NEG |
| ccrB-1 | Cassette Chromosome Recombinase B, type 1 | NEG | NEG | NEG | NEG | NEG | NEG | NEG | NEG |
| ccrB-2 | Cassette Chromosome Recombinase B, type 2 | **POS** | **POS** | **POS** | **POS** | **POS** | **POS** | **POS** | **POS** |
| ccrB-3 | Cassette Chromosome Recombinase B, type 3 | NEG | NEG | NEG | NEG | NEG | NEG | NEG | NEG |
| ccrB-4 | Cassette Chromosome Recombinase B, type 4 | NEG | NEG | NEG | NEG | NEG | NEG | NEG | NEG |
| ccrC | Cassette Chromosome Recombinase C | NEG | NEG | NEG | NEG | NEG | NEG | NEG | NEG |
| merA | Mercuric Reductase (SCCmec Type III) | NEG | NEG | NEG | NEG | NEG | NEG | NEG | NEG |
| merB | Alkylmercury Lyase (SCCmec Type III) | NEG | NEG | NEG | NEG | NEG | NEG | NEG | NEG |
| kdpA-SCC | Potassium-transporting ATPase A chain | NEG | NEG | NEG | NEG | NEG | NEG | NEG | NEG |
| kdpB-SCC | Potassium-transporting ATPase B chain | NEG | NEG | NEG | NEG | NEG | NEG | NEG | NEG |
| kdpC-SCC | Potassium-transporting ATPase C chain | NEG | NEG | NEG | NEG | NEG | NEG | NEG | NEG |
| kdpD-SCC | Sensor Histidine Kinase | NEG | NEG | NEG | NEG | NEG | NEG | NEG | NEG |
| kdpE-SCC | KDP Operon Transcriptional Regulatory Protein | NEG | NEG | NEG | NEG | NEG | NEG | NEG | NEG |
| plsSCC-COL | Plasmin-Sensitive Surface Protein | NEG | NEG | NEG | NEG | NEG | NEG | NEG | NEG |
| Q9XB68-dcs | Hypothetical Protein Historical name: CN050 Synonyms:dcs | NEG | **AMB** | NEG | NEG | NEG | NEG | NEG | NEG |
| xylR | Pseudogene of Xylose Repressor | NEG | NEG | NEG | NEG | NEG | NEG | NEG | NEG |
| **Capsule/Biofilm** |  |  |  |  |  |  |  |  |  |
| capsule-1 | Capusle Type 1 | NEG | NEG | NEG | NEG | NEG | NEG | NEG | NEG |
| capsule-5 | Capusle Type 5 | NEG | NEG | NEG | NEG | NEG | NEG | NEG | NEG |
| capsule-8 | Capusle Type 8 | **POS** | **POS** | **POS** | **POS** | **POS** | **POS** | **POS** | **POS** |
| capH1 | Capsular Polysaccahride Synthesis Enzyme CapH Capsule Type 1 | NEG | NEG | NEG | NEG | NEG | NEG | NEG | NEG |
| capJ1 | O-Antigen Poymerase CapJ Capsule Type 1 | NEG | NEG | NEG | NEG | NEG | NEG | NEG | NEG |
| capK1 | Capsular Polysaccahride Biosynthesis Protein CapK Capsule Type 1 | NEG | NEG | NEG | NEG | NEG | NEG | NEG | NEG |
| capH5 | Capsular Polysaccahride Synthesis Enzyme CapH Capsule Type 5 | NEG | NEG | NEG | NEG | NEG | NEG | NEG | NEG |
| capJ5 | O-Antigen Poymerase CapJ Capsule Type 5 | NEG | NEG | NEG | NEG | NEG | NEG | NEG | NEG |
| capK5 | Capsular Polysaccahride Biosynthesis Protein CapK Capsule Type 5 | NEG | NEG | NEG | NEG | NEG | NEG | NEG | NEG |
| capH8 | Capsular Polysaccahride Synthesis Enzyme CapH Capsule Type 8 | **POS** | **POS** | **POS** | **POS** | **POS** | **POS** | **POS** | **POS** |
| capI8 | Capsular Polysaccahride Synthesis Enzyme CapI Capsule Type 8 | **POS** | **POS** | **POS** | **POS** | **POS** | **POS** | **POS** | **POS** |
| capJ8 | O-Antigen Poymerase CapJ Capsule Type 8 | **POS** | **POS** | **POS** | **POS** | **POS** | **POS** | **POS** | **POS** |
| capK8 | Capsular Polysaccahride Biosynthesis Protein CapK Capsule Type 8 | **POS** | **POS** | **POS** | **POS** | **POS** | **POS** | **POS** | **POS** |
| icaA | Intercellular Adhesion Protein A | **POS** | **POS** | **POS** | **POS** | **POS** | **POS** | **POS** | **POS** |
| icaC | Intercellular Adhesion Protein C | **POS** | **POS** | **POS** | **POS** | **POS** | **POS** | **POS** | **POS** |
| icaD | Biofilm PIA Synthesis Protein D | **POS** | **POS** | **POS** | **POS** | **POS** | **POS** | **POS** | **POS** |
| Bap | Surface Protein Involved in Biofilm Formation | NEG | NEG | NEG | NEG | NEG | NEG | NEG | NEG |
| **MSCRAMMs/Adhesion Factors** |  |  |  |  |  |  |  |  |  |
| Bbp | Bone Sialoprotein-Binding Protein | **POS** | **POS** | **POS** | **POS** | **POS** | **POS** | **POS** | **POS** |
| clfA | Clumping Factor A | **POS** | **POS** | **POS** | **POS** | **POS** | **POS** | **POS** | **POS** |
| clfB | Clumping Factor B | **POS** | **POS** | **POS** | **POS** | **POS** | **POS** | **POS** | **POS** |
| Cna | Collagen Binding Adhesion | NEG | NEG | NEG | **POS** | **POS** | **POS** | **POS** | NEG |
| Ebh | Cell Wall Associated Fibronectin Binding Protein | NEG | NEG | **POS** | **POS** | **POS** | **POS** | **POS** | **POS** |
| Eno | Enolase, Phosphopyruvate Hydratase | **POS** | **POS** | **POS** | **POS** | **POS** | **POS** | **POS** | **POS** |
| Fib | Fibrinogen Binding Protein | **POS** | **POS** | **POS** | **POS** | **POS** | **POS** | **POS** | **POS** |
| ebpS | Cell Wass Associated | **POS** | **POS** | **POS** | **POS** | **POS** | **POS** | **POS** | **POS** |
| fnbA | Fibronectin Binding Protein A | **POS** | **POS** | **POS** | **POS** | **POS** | **POS** | **POS** | **POS** |
| fnbB | Fibronectin Binding Protein B | **POS** | **POS** | **POS** | **POS** | **POS** | **POS** | **POS** | **POS** |
| Map | Major Histocompatibility Complex Class II Analog Protein | NEG | NEG | **POS** | **POS** | **POS** | **POS** | **POS** | **POS** |
| sdrC | Ser-Asp Rich Fibrinogen Binding, Bobe Sialoprotein Binding Protein C | **POS** | **POS** | **POS** | **POS** | **POS** | **POS** | **POS** | **POS** |
| sdrD | Ser-Asp Rich Fibrinogen Binding, Bobe Sialoprotein Binding Protein D | **POS** | **POS** | **POS** | **POS** | **POS** | **POS** | **POS** | **POS** |
| Vwb | Willebrand Factor Binding Protein | **POS** | **POS** | **POS** | **POS** | **POS** | **POS** | **POS** | **POS** |
| sasG | S aureus Surface Protein G | NEG | NEG | **POS** | **POS** | **POS** | **POS** | **POS** | **POS** |
| **Immunovasion and Miscellaneous** |  |  |  |  |  |  |  |  |  |
| isaB | Immunodominant Antigen B | **POS** | **POS** | **POS** | **POS** | **POS** | **POS** | **POS** | **POS** |
| mprF | Probable Lysylphosphatidylglycerol Synthetase | NEG | NEG | **POS** | **POS** | **POS** | **POS** | **POS** | **POS** |
| isdA | Haem ?Transferrin Binding Protein | **POS** | **POS** | **POS** | **POS** | **POS** | **POS** | **POS** | **POS** |
| 1mrP | Putative Transporter Protein | **POS** | **POS** | **POS** | **POS** | **POS** | **POS** | **POS** | **POS** |
| Q2YUB3 | Multidrug Resistance Protein | NEG | NEG | NEG | NEG | NEG | NEG | NEG | NEG |
| hsdS1 | Site Specific Deoxyribonuclease Subunit Type 1 | NEG | NEG | **POS** | NEG | NEG | NEG | NEG | **POS** |
| hsdS2 | Site Specific Deoxyribonuclease Subunit Type 2 | NEG | NEG | NEG | **POS** | **POS** | **POS** | **POS** | **NEG** |
| hsdS3 | Site Specific Deoxyribonuclease Subunit Type 3 | **POS** | **POS** | **POS** | **POS** | **POS** | **POS** | **POS** | **POS** |
| hsdSx | Site Specific Deoxyribonuclease Subunit Type X | NEG | NEG | **POS** | **POS** | **POS** | **POS** | **POS** | NEG |
| Q2FXCO | Q2FXCO | NEG | NEG | NEG | **POS** | **POS** | **POS** | **POS** | **POS** |
| Q7A4X2 | Hypothetical protein next to entG | **POS** | **POS** | NEG | NEG | NEG | NEG | NEG | NEG |
| hysA1 | Hyaluronate Lyase A1 | **POS** | **POS** | **POS** | **POS** | **POS** | **POS** | **POS** | **POS** |
| hysA2 | Hyaluronate Lyase A2 | **POS** | **POS** | **POS** | **POS** | **POS** | **POS** | **POS** | **POS** |
| **set/ssl Genes** |  |  |  |  |  |  |  |  |  |
| setC | Staphylococcal Superantigen-Like Protein C | **POS** | **POS** | **POS** | **POS** | **POS** | **POS** | **POS** | **POS** |
| set6 | Staphylococcal Superantigen-Like Protein 6/SSL1 | NEG | **POS** | **POS** | **POS** | **POS** | **POS** | **POS** | **POS** |
| set7 | Staphylococcal Superantigen-Like Protein 7/SSL2 | NEG | NEG | **POS** | **POS** | **POS** | **POS** | **POS** | **POS** |
| set8 | Staphylococcal Superantigen-Like Protein 8/SSL3 | NEG | NEG | **POS** | **POS** | **POS** | **POS** | **POS** | **POS** |
| set9 | Staphylococcal Superantigen-Like Protein 9/SSL4 | NEG | NEG | **POS** | **POS** | **POS** | **POS** | **POS** | **POS** |
| set3 | Staphylococcal Superantigen-Like Protein 3/SSL5 | NEG | NEG | **POS** | **POS** | **POS** | **POS** | **POS** | **POS** |
| set21 | Staphylococcal Superantigen-Like Protein 21/SSL6 | **POS** | **POS** | **POS** | **POS** | **POS** | **POS** | **POS** | **POS** |
| set1 | Staphylococcal Superantigen-Like Protein 1/SSL7 | NEG | NEG | **POS** | **POS** | **POS** | **POS** | **POS** | **POS** |
| set12 | Staphylococcal Superantigen-Like Protein 12/SSL8 | NEG | NEG | **POS** | **POS** | **POS** | **POS** | **POS** | **POS** |
| set5 | Staphylococcal Superantigen-Like Protein 5/SSL9 | NEG | NEG | **POS** | **POS** | **POS** | **POS** | **POS** | **POS** |
| set4 | Staphylococcal Superantigen-Like Protein 4/SSL10 | NEG | NEG | **POS** | **POS** | **POS** | **POS** | **POS** | **POS** |
| set2 | Staphylococcal Superantigen-Like Protein 2/SSL11 | NEG | NEG | NEG | **POS** | **POS** | **POS** | **POS** | NEG |
| setB3 | Staphylococcal Superantigen-Like Protein B3 | NEG | NEG | **POS** | **POS** | **POS** | **POS** | **POS** | **POS** |
| setB2 | Staphylococcal Superantigen-Like Protein B2 | NEG | NEG | **POS** | **POS** | **POS** | **POS** | **POS** | **POS** |
| setB1 | Staphylococcal Superantigen-Like Protein B1 | NEG | NEG | **POS** | **POS** | **POS** | **POS** | **POS** | **POS** |
